# Understanding perspectives of HIV/AIDS affected households on food and nutrition interventions and social protection programmes in Zimbabwe

**DOI:** 10.3389/fnut.2024.1358203

**Published:** 2024-06-24

**Authors:** Kembo George, Mavis Precious Dembedza, Macheka Lesley

**Affiliations:** ^1^Food and Nutrition Council of Zimbabwe, Harare, Zimbabwe; ^2^Centre for Innovation and Industrialisation, Marondera University of Agriculture Science and Technology, Marondera, Zimbabwe

**Keywords:** HIV/AIDS, food and nutrition interventions, programming, lived experiences, Zimbabwe

## Abstract

**Introduction:**

The study was aimed at understanding the needs and perspectives of HIV affected households on food and nutrition security intervention programmes.

**Methods:**

The study used qualitative methods that include focus groups discussions and key informant interviews to solicit for lived experiences of People Living With HIV (PLWHIV).

**Results:**

The results revealed that intervention programmes by both government and development partners (donors) can be divided into four (4) categories: food and nutrition security, livelihood, health, and social protection. Interventions that targeted health included the provision of HIV antiretroviral drugs to PLWHIV and counselling to both PLWHIV and affected persons. Intervention programmes targeted at social protection included provision of food aid and cash transfers.

**Discussion:**

The recommendations based on the research findings are that intervention programmes should focus more on resilience building as a way of building capacity of PLWHIV. This way, sustainability of intervention programmes is improved. As such, it is important to ensure, through policy, that all intervention programmes have a component of capacity building to improve resilience of participants and programme sustainability. Furthermore, there is a need to improve targeting for beneficiaries of intervention programmes and clearly define the “vulnerable” group.

## 1 Introduction

The interconnections between HIV/AIDS, food insecurity, and undernutrition are increasingly understood, leading to a surge of interventions. The impact of HIV/AIDS on food insecurity has been well-documented in Africa and manifests through the debilitation of the most productive household members, decreased household economic capacity, decreased household agricultural output, and increased caregiver burden (1–3). Moreso, observational studies suggest that food insecurity is associated with increased HIV transmission risk behaviours and decreased access to HIV treatment and care (4–9). There is now a plethora of evidence on how to mitigate the food security and nutrition dimensions of the epidemic.

According to Aberman et al. (10) nutrition supplementation and safety nets in the form of food assistance and livelihood interventions have potential in certain contexts to improve food security and nutrition outcomes in an HIV/AIDS context. In addition, several policies and frameworks have been advanced to aid the integration of food and nutrition programs into a comprehensive response against the AIDS epidemic (11, 12). Examples of programmatic models aiming to improve food security and nutrition in an HIV context include (10, 11, 13) (i) nutrition supplementation interventions targeted at undernourished People Living With HIV/AIDS (PLWHIV), (ii) safety nets targeted at HIV-affected households and individuals to improve household food security, mitigate the impact of HIV, and (iii) livelihood interventions targeted at PLWHIV households or communities affected by the AIDS epidemic. With increasing recognition of the importance of integrating food security and nutrition interventions into HIV care and treatment programs (13), targeted food assistance have been used by many organisations to assist the HIV-affected individuals and households (14). Food assistance programs providing a household food basket targeted to food insecure PLWHA and their households are the most ubiquitous of these program modalities (15).

Several quantitative analyses have been conducted in Zimbabwe to establish the food and nutrition status of HIV-affected households (16–18). Major findings from these studies are that (i) HIV-affected households are more likely to be cereal food insecure than the not affected households, (ii), female headed households are more likely to be HIV-affected as compared to male headed households and are more vulnerable to food and nutrition insecurity as compared to their male counterparts, and (iii) HIV-affected households are at a disadvantage with regards to social protections and they are not a priority in most of the social protection programmes. However, limited qualitative studies have been done to explain and understand the observed results from quantitative studies. Firsthand information from qualitative studies and lived experiences from the affected population will help to unravel the underlying factors to findings from quantitative studies. Complimenting findings from quantitative studies with the lived experiences can result in better programming of the intervention programme (19, 20). Quantitative studies will help capture the voices of the affected and help get in-depth insights into the needs and perceptions of HIV-affected households regarding food and nutrition intervention programmes.

The main objective of this study was to ascertain the social protection needs of HIV-affected households in selected districts in Zimbabwe. Specifically, the study was aimed at getting in-depth insights into, (i) the views and perspectives on the food and nutrition security, livelihoods and social protection needs of HIV-affected households in selected districts in Zimbabwe, (ii) the criteria used in social protection targeting by the different social protection providers, such as government, development partners, civil society and social groups, and (ii) the preferred food and nutrition interventions and social protection programmes by the HIV-affected households.

## 2 Methodology

### 2.1 Study sites

**Figure 1:**
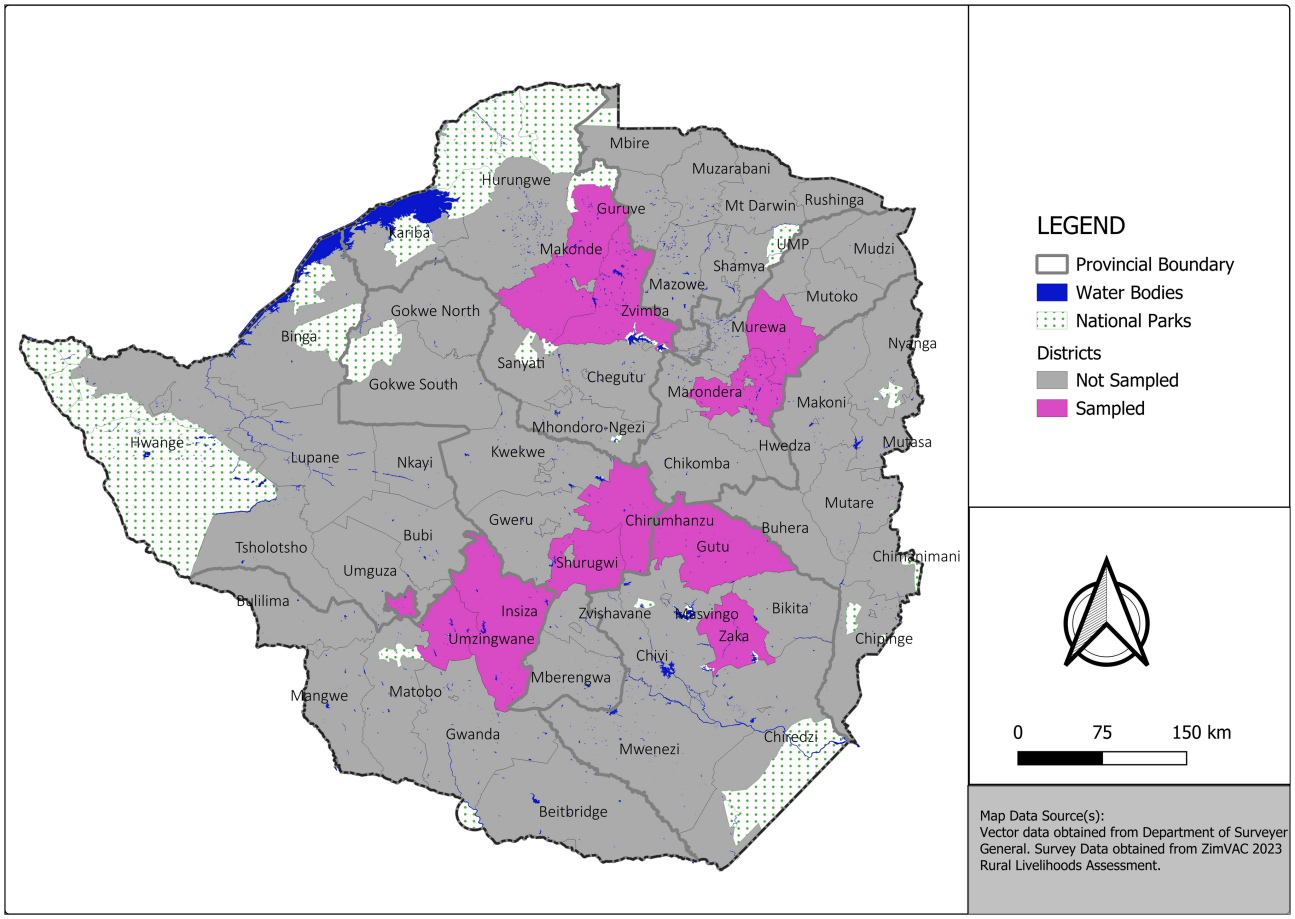
Map showing sampled districts.

The study was conducted in six provinces in Zimbabwe, and in each province, two districts were selected. The selected provinces and districts included Magwegwe and Nkulumane districts in Bulawayo province, Insiza and Umzingwane districts in Matabeleland South province, Zaka and Gutu districts in Masvingo province, Churumhanzu and Shurugwi districts in Midlands province, Zvimba and Makonde districts in Mashonaland West province, and Marondera and Murehwa districts in Mashonaland East province (Figure 1). Purposive sampling was conducted using data from the National Aids Council, which identified the provinces and districts where programs aimed at HIV/AIDS affected households were being implemented by the government and/or NGOs.

### 2.2 Data collection

Data was collected through Focus Group Discussions (FGDs) and Key Informant Interviews (KIIs). Data was collected from the 12th to the 14th of December 2022. Data was collected using three teams, each consisting of one supervisor and two enumerators. Each team collected data from two provinces, which amounts to four districts. The enumerators were selected from the database of enumerators who had previously participated in at least three ZimVAC assessments; therefore, they had experience in data collection.

#### 2.2.1 Focus group discussions

Two (2) FDGs were conducted in each district, giving a total of four (4) FDGs per province and overall, 12 FDGs for the study. The number of participants in each FGD varied from eight (8) to 12 individuals. Participants were identified through already existing structures of organisations working with HIV-affected households and support groups. National Aids Council (NAC) assisted with participant identification and mobilisation for FGDs in all the sampled districts. The Ministry of Public Service Labour and Social Welfare and other local NGOs working in the sampled districts were also consulted. The discussions were guided by a semi-structured questionnaire which solicited for the following information: (i) participation in intervention programmes, (ii) inclusion in designing and developing the intervention programmes, (iii) coping strategies and resilience building activities, and (iv) recommendations by participants on how to improve the intervention programmes. These four thematic areas discussed during the FGDs are the focus areas of most intervention programmes by government and NGOs in Zimbabwe.

#### 2.2.2 Key informant interviews (KIIs)

Semi-structured in-depth interviews with selected key informants were used to collect data (see Annex 2). The key informants were drawn from key stakeholders providing social support and implementing HIV intervention programmes in Zimbabwe such as the Department of Social Protection and National Aids Council, among others. KIIs were conducted as a way of triangulation and to provide an important supplement to the qualitative interviews conducted through FGDs. Information solicited from the KIIs was mainly on the historical and contemporary perspectives on social protection and food and nutrition security programming toward the HIV-affected population, including perspectives based on their familiarity with the health, legislative and welfare systems governing individuals’ lives in the communities they work with.

### 2.3 Data analysis

The responses from both KIIs and FGDs were transcribed verbatim in English. The research team used an inductive thematic analysis to guide the coding process and identified emerging themes and methodology described by Braun and Clarke (21) was followed. Each of the transcripts was manually coded using a pre-defined codebook. Codes generated from two researchers who did the coding were compared for differences and similarities and to evaluate inter-coder reliability. In addition, the Consolidated criteria for reporting qualitative research (COREQ) (22) were followed to ensure quality in reporting the study.

### 2.4 Ethical issues

Written permission to conduct the study was obtained from the National Aids Council and ethical approval of the protocol was obtained from the Marondera University of Agricultural Sciences and Technology Ethical Committee. Written informed consent was obtained from all study participants.

## 3 Results

### 3.1 Demographics of focus group discussions

**Figure 2:**
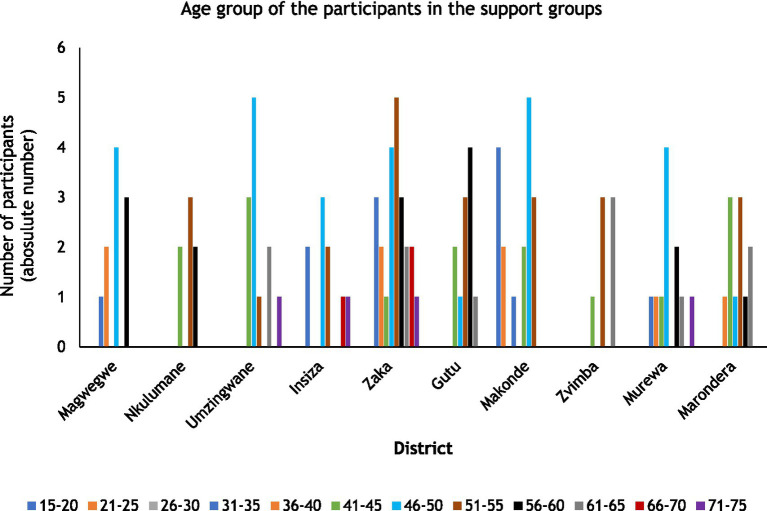
Age group of participants in the support groups. This figure based on data from 6 districts only. In the other districts, participants were not willing to share information on their age.

A total of 143 people participated in the FGDs, with the highest number of participants (27) recorded in Shurugwi and the lowest (7) recorded in Zvimba and Nkulumane districts (Table 1). Furthermore, the results in Table 1 revealed that females (78.3%) dominated the support groups, while males constituted only 21.7%. In the sampled districts, all support groups had more females than males, except for Shurugwi where more men (55.6%) than females (44.4%) participated. The results revealed that most of the participants, 53.8% (77), in the sampled support groups were HIV positive while 30.1% (43) were affected. Makonde district was the only district with participants that were under the age of 26 years (35.3%). As such, the views of the youths were captured in this study. Regarding age group, results presented in Figure 2 show that most (23.9%) of the participants were within the age group 46–50 years, 19.5% in the 51–55 years age group and 16.7% were in the 41–45 and 56–60 age group.

**Table 1:** Demographics of FDGs participants.

Province	District	Gender of FDG participants			Age of FDG participants		Status	
		Male	Female	Total	Average age	Range	PLWHIV	Affected
Bulawayo	Magwegwe	1	9	10	46.4	30–58	9	1
	Nkulumane	0	10	10	50.1	42–56	10	0
Matabeleland South	Insiza	4	6	9	49.9	33–72	10	0
	Umzingwane	1	11	12	51.1	43–73	12	0
Masvingo	Zaka	0	10	10	*	*	9	1
	Gutu	1	10	11	*	*	8	3
Midlands	Churumhanzu	0	10	10	*	*	9	1
	Shurugwi	15	12	27	*	*	26	1
Mashonaland West	Zvimba	2	5	7	56.1	44–65	7	0
	Makonde	1	16	17	37.6	18–53	0	17
Mashonaland East	Marondera	4	9	13	50.6	38–62	13	0
	Murehwa	3	7	10	50.0	31–72	10	0
	Total	31	112	143			77	43

*Data not available as participants were not willing to divulge the information.

### 3.2 Intervention programmes

Table 2 shows the different types of HIV sensitive intervention programmes implemented by both government and NGOs. The interventions can be divided into four (4) categories: food and nutrition security, livelihood, health and social protection. Common interventions under the food and nutrition security category included support with agricultural inputs for community and/ or nutrition gardens, support with small livestock (poultry and rabbit) production and training on smart agriculture.

**Table 2:** Intervention programmes.

Category	Type of intervention
1. Food and nutrition security	*Government* Cash transfer Agricultural inputs for community gardens Small livestock production (poultry and rabbit production) Smart agriculture training – Pfumvudza Nutrition gardens Nutrition education *Non-Governmental Organisations (NGOs)* Food aid Supplementary feeding – meals Capacity building projects: Community Garden and self-help projects like pine gel making Provision of inputs for horticulture Nutrition gardens Nutrition education
2. Livelihood	*Government* Capacity building: training on welding, floor polish making, sanitizer dispensers and broiler rearing. Supported formation of ISAL groups *Non-Governmental Organisations (NGOs)* Supported formation of ISAL groups Capacity building: E.g., training on manufacturing dish washing soap, bee keeping
3. Health	*Government* Support to HIV positive pregnant women ART mobile clinic once a month HIV/TB campaigns offering co-infection services Support groups formed by AIDS Activists Counselling of PLWHIV to minimise withdrawal Offers prevention services including condoms *Non-Governmental Organisations (NGOs)* Free HIV Counselling Services Psychosocial support on medication adherence using Community HIV/AIDS Support Agents (CHASA) Community Outreach Agents (COA) Community Referral Facilitators (CRF) Community Adherence and Treatment Support (CATS) and Peer Educators TB care and treatment Cancer screening for pregnant women Provision of bicycles for community health workers in HIV programs
4. Social protection	*Government* Food aid (50 kg per household) Cash transfer Basic Education Assistance Module (BEAM) programme *Non-Governmental Organisations (NGOs)* Cash transfer Supported formation of ISAL groups Food aid

As for interventions targeting to improve the livelihood of the HIV-affected households, capacity building through provision of trainings in technical skills, such as welding, floor polish marking, support with small grants to start small businesses, and support to form Internal Savings and Lending (ISAL) groups were the most common intervention programmes implemented by both government and NGOs.

Several interventions targeting health were highlighted by the participants. These include provision of HIV antiretroviral drugs to the PLWHIV and counselling to both PLWHIV and the affected persons (Table 2). Lastly, Table 2 shows that some of the intervention programmes were targeted at social protection and examples of such programmes include provision of food aid and cash transfers.

### 3.3 Participation in available intervention programmes

Table 3 presents the findings from the FGDs. The results show that intervention programmes were usually not sensitive to the actual needs of the HIV-affected households. More so, the results indicate that the coverage in terms of geographical spread and number of beneficiaries was low. The participants also indicated that the beneficiary selection criterion used did not include HIV-affected households. Moreover, the participants revealed that in some cases, HIV status was not regarded as a vulnerability, hence HIV-affected households were not being targeted. According to the participants, the line of reasoning for not including PLWHIV and the affected was that being HIV positive or affected does not stop one from working and generating income or produce own food. In some cases, stigmatisation was indicated as a barrier to participation of PLWHIV or the affected in the intervention programmes.

**Table 3:** Common responses regarding inclusion of the HIV-affected in intervention programmes.

Question	Responses
Sensitivity to HIV-affected households	General criteria used targets the vulnerable households, and unfortunately, interpretation of ‘vulnerable’ excludes the HIV-affected household in some cases. Not sensitive and usually what is agreed at inception is not adhered to during implementation, especially the agreed beneficiary selection criteria. The programmes are sensitive because the individuals living with HIV spearhead the programmes. Hence, they will be motivated to see the programmes through.
Easy of participation in intervention programmes	It is difficult as most programmes do not recognise HIV-affected households/people as vulnerable. Community is polarised contributing to some members not participating and the HIV-affected being segregated.
Barriers to participation in intervention programmes	Selection criteria sometimes exclude the HIV-affected. Polarisation of community. Politics – wrong political affiliation. Corruption and bias by the community representatives. Information on the intervention programmes is sometimes not cascaded to everyone. Some programmes require benefactors to pay subscriptions which they do not afford and in return benefit nothing.
Involvement in implementation of intervention programmes	Communities are involved and consulted, and all partners have sensitization meeting before they embark on their intervention programmes and communities are consulted for their input. However, in some cases the deserving beneficiaries are excluded during programme implementation.
Inclusivity of the intervention programmes	Most selection criteria of the intervention programmes are based on gender, age etc. Hence, not all categories of people are selected included. In some cases, men only are recognised as household heads and single mothers are left out. The youth are also neglected because only the elderly are considered to be more vulnerable.
Availability of platforms for community/beneficiary feedback	There are no mechanisms for communities of beneficiaries to give feedback on the performance of the programmes. There are no formal mechanisms for capturing the complaints of beneficiaries. Social welfare offices sometimes do not act on the concerns of the community. There are suggestion boxes and sometimes service providers share contact numbers for queries and comments.

*The programmes are not sensitive mainly because the criteria used targets the vulnerable households, and unfortunately, interpretation of ‘vulnerable’ excludes the HIV-affected household in some cases.*

However, participants reported a significant positive development in health interventions. This is because the programmes specifically targeted PLWHIV and the affected. Beneficiaries indicated that health interventions were specific to the needs of PLWHIV.

Unfortunately, a plethora of barriers still exist and prevent the HIV-affected households from accessing some of the intervention programmes. The respondents indicated that in some programmes, the selection criteria were not sensitive to the needs of PLWHIV, and other challenges include polarisation of the community, lack of adequate information on the intervention programmes, corruption leading to the underserving benefiting, and worse, some programmes require benefactors (the vulnerable) to pay subscriptions, which they do not afford. On a positive note, most of the participants indicated that they are being consulted through project inception meetings and involved in beneficiary selection. However, in some cases their input is not implemented.

### 3.4 Coping strategies and resilience building

The results presented in Table 4 show the coping strategies being implemented by HIV-affected households in response to the challenges they face. The participants highlighted shortages of food and drinking water and lack of dietary diversity in the food provisions from Social Welfare and NGOs as some of the challenges they encounter. Challenges that include limited resources to sustain nutrition gardens and limited income generating projects were also raised.

**Table 4:** Coping strategies and resilience building.

Question	Response
Main challenges pertaining to food and nutrition security	Food shortages Shortage of drinking water Poor food consumption patterns (no dietary diversification) Food basket being provided through Social Welfare does not meet dietary needs Limited income generating programmes Limited resources to sustain nutrition gardens Lack of training in managing projects Limited resources for capacitation and training of communities
Consumption coping strategies	Purchase food on credit Send household members to beg Skip entire days without eating Borrow food or rely on help from a friend or relative Reduction in number of meals eaten in a day Reducing meal portions Finding alternative foods like wild fruits that are in season
Livelihood coping strategies	Rely on casual labour and menial jobs Youths turn to negative social vices – prostitution and crime to get money for food Selling firewood Child labour as children go to look for piece jobs Withdrawal of children from school Rely on casual labour for food
Sustainability of the programmes	No, some of the programmes do not leave the community empowered. Project assets should be left with the community or support groups so that they are able to continue implementing the interventions. Yes, some of the programmes such as tin smithing and detergent making empower the community. Yes, because the community oversees the projects themselves with limited help. Yes, HIV-affected households were trained and capacitated with knowledge to run ISALS

Faced with these challenges, the HIV-affected households employed several coping strategies for them to survive. Some of the coping strategies relate to consumption and strategies employed include purchase food on credit, begging for food, skipping the entire days without eating, borrowing food, or rely on help from a friend or relative, findings alternative option, such as wild fruits, took up casual labour and menial jobs, and some engaged in selling firewood to raise income. Unfortunately, some youths employed negative coping strategies such as prostitution and crime. These are high risk strategies that fuel the spread of HIV.

*Teenagers now resort to prostitution, theft and drug abuse to get money for food. Those engaging in prostitution, they charge as little as USD 2 to attract more clients here at the local bar.*

*I have used sex as a source of income to buy food. After collecting my medication, I solicited for clients at the nearby bar and sometimes I can get USD 20 which I use for transport and groceries*.

During the FGDs, mixed views were generated from the participants regarding sustainability of the intervention programmes. Some participants indicated that the programmes were sustainable, whiles others highlighted that the programmes are not sustainable. Sustainability in the context of this study was considered as the extent to which the programmes empowered and capacitated the community or targeted group, allowing for continuity of the programmes even after the end of the funding from government or donors.

*No, the interventions programmes being implemented are not sustainable as they do not leave the community empowered. Project assets should be left with the community or support groups after the project ends so that the beneficiaries can continue implementing the interventions.*

On the positive side, participants in Midlands province reported that some NGOs have assisted HIV-affected households establish viable income generative projects, which are sustainable.

*Yes, Midlands AIDS Service Organisation assisted us to establish a support group that has now established a sustainable nutrition garden.*

### 3.5 Recommended intervention programmes and activities

Table 5 presents the findings on the intervention programmes preferred by the participants, i.e., HIV-affected households/individuals. Under food and nutrition interventions, the participants indicated the following programmes should be prioritised: nutrition gardens and training on low input gardening techniques, small livestock production (poultry and rabbits), cash transfer to support access to balanced diets, food assistance and supplementary feeding for the affected households, training on processing and value addition of traditional food, value addition equipment to provide vegetables all year around, among other programmes.

**Table 5:** Recommended intervention programmes and activities.

Intervention programmes	Activities proposed
Food and nutrition security	Nutrition gardens and training on low input gardening techniques Small livestock production (poultry and rabbits). Cash transfer to support access to balanced diets. Food assistance and supplementary feeding for the affected households. Training on processing and value addition of traditional food. Drilling of boreholes to improve water access. Improve access to improve and diversified diets. Value addition equipment to provide vegetables all year around. Establishment of orchards to provide fruits all year around. Land for farming both gardening and livestock agriculture (also promotes self-sufficiency).
Health	Medical assistance for PLWHIV, i.e., improve access to drugs for opportunistic infections. Improve access to health facilities. Increase outreach programmes to improve access to health services. Scrapping off user fees for PLWHIV and affected households.
Social protection	HIV sensitive food baskets targeted at HIV support groups. Distribution of food commodities. Policies to promote equality and de-stigmatisation. Cash transfers. Medical treatment assistance. Care for child and orphan headed households.
Livelihoods	Provide capital and or provision of small grants/loans for self-help income generating projects. Provide more land for farming. Come up with programmes that teach independence and self-sufficiency. Support small livestock rearing projects to complement income and diet. Provide training on project management.

Regarding health-related interventions, the most proposed intervention programme by the participants was free medical care for opportunistic infections and for treatment of side effects of ARVs.

*We propose medical assistance for PLWHIV. There are a lot of opportunistic infections that come with being HIV positive and currently we are not getting free medical care. Moreover, in some cases ARVs have side effects, which are known, and we propose free medical care for treatment of these side effects.*

### 3.6 Findings from key informant interviews

**Figure 3:**
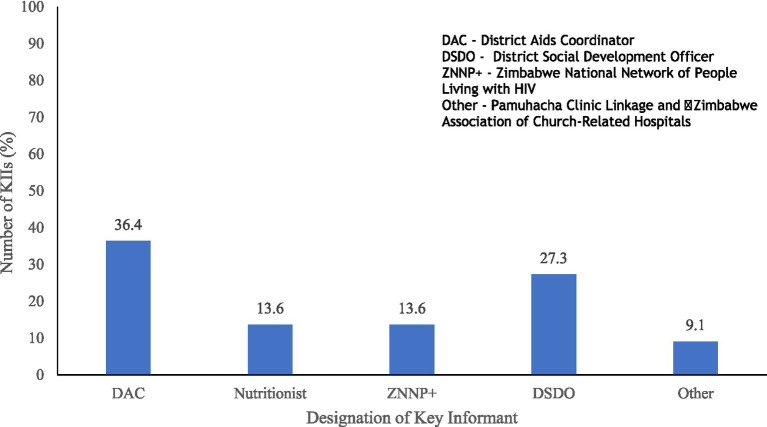
Designation of key informants interview respondents.

A total of 19 key informants were interviewed to solicit information on available intervention programmes targeting the HIV-affected households, inclusivity, and sustainability of these programmes. The results in Figure 3 show that the KIs were drawn from various sectors and most (36.8%) were District AIDS Coordinators, 21.1% were District Development Social Officers, and Nutritionist and Zimbabwe National Network of People Living with HIV Coordinators constituted 15.8% of the KIs interviewed.

The findings from the KIIs corroborate with the findings from the FDGs. The KIIs gave some indepth insights into the reasons for some of the findings from the FGDs. For example, the District Social Development Officer from Zvimba district explained on the coverage of intervention programmes:

*Coverage of intervention programmes targeting the HIV-affected households is almost 100%. However, some of the affected are not participating in the intervention programmes because they fear stigmatisation from the community and family members if they disclosure their HIV status.*

Regarding the inclusion criteria to be a beneficiary in intervention programmes, it was reported that:

*The main criterion used in interventions targeting PLWHIV is that one must be living with HIV. The participants are identified using records from health facilities, e.g., records of persons participating in antiretroviral drugs programmes. However, in some programmes that target the vulnerable (such as food aid), PLWHIV are sometimes not classified as a vulnerable group, therefore they are not selected as beneficiaries.*

The key informants indicated that the intervention programmes are sustainable:

*The projects are people driven and the community members are active. However, to a lesser extent the intervention programmes are not sustainable because to be able to identify needs better the programming strategy used should be bottom up.*

The impact and sustainability of the intervention programmes was applauded by the key informants.

*Yes, the intervention programmes have impact and are sustainable because most of these programmes use the multi-sectoral approach and there are many players. The programme involves communities and community participation making these programmes sustainable. The impact of the intervention programmes is evident as the HIV/AIDS prevalence has gone down over the past few years. However, lack of funds and resources has greatly hampered the sustainability of some of the intervention programmes.*

## 4 Discussion

The presented study is one of the few qualitative studies that have managed to capture the views of the different groups of PLWHIV in Zimbabwe, that is, men, women, and the youths. Previous studies have either focused on women (23), adults (18), or children (16) and few cross-sectional studies considered all the ages groups and sex in one study. The low participation of men in intervention programmes reported in the presented study is common across other studies presented in literature. According to Mantell et al. (24) and Okal et al. (25), concerns about stigma and privacy were perceived to be the primary reason for men’s non-participation.

Findings that capacity building through provision of trainings in technical skills, such as welding, floor polish marking, support with small grants to start small businesses, and support to form Internal Savings and Lending (ISAL) groups, was the most common intervention implemented by both government and NGOs corroborate previous studies (26, 27) in which ISALS were found to contribute to improved financial wellbeing of the affected individuals and households.

However, our study revealed that some of the intervention programmes were not sensitive to the actual needs of the HIV-affected households. This finding resonate with results from a study by Mokomane et al. (28) which revealed that social protection policies and social assistance programmes in Southern Africa do not specifically target HIV issues or people living with, at risk of, or affected by HIV. Rather, the programmes tend to be inclusive of the vulnerabilities of various populations including PLWHIV. According to van der Wal et al. (29), to be HIV-sensitive, intervention programmes should be designed around the interests, needs and vulnerability of the affected, adapted to local implementation contexts, and include life skills to capacitate those affected.

The finding that some of the PLWHIV, especially youths, employed negative coping strategies such as prostitution and crime is unfortunate as these coping strategies fuel the spread of HIV. Similar findings were reported in literature and the use of negative strategies was found to be a reaction to the threat of food insecurity (30), unemployment, financial stress, fear of stigma, disclosure worries and concerns, and limited social support (31). Dake et al. (32) and Laar et al. (30) emphasised on the importance of a good social support and improved sensitivity and inclusivity of intervention programmes as way to reduce use of negative coping strategies by the affected.

The weakness of the study is that it was cross-sectional study and therefore it was difficult to assess whether some of the findings, e.g., poor coping strategies, existed before changes in some of the independent variables occurred.

## 5 Conclusion and policy recommendations

The study sought for lived experiences to understand the perspectives and needs of HIV-affected households. Firsthand information and lived experiences from the affected generated and presented in paper can be used inform decisions, policies and programming of intervention programmes. The recommendations based on the findings are that intervention programmes should focus more on resilience building as a way of building capacity of PLWHIV and the affected population. This way, sustainability of implemented programmes is improved. As such, it is important to ensure, through policy, that all intervention programmes have a component of capacity building to improve resilience of participants and programme sustainability. Furthermore, there is a need to improve targeting for beneficiaries of intervention programmes and clearly define the “vulnerable” group. Lastly, there is need to purposely target men in campaigns for voluntary participation in HIV intervention programmes. The campaigns should also be targeted toward the public to diffuse stigmatisation of the affected by the society.

## Data availability statement

The raw data supporting the conclusions of this article will be made available by the authors, without undue reservation.

## Ethics statement

The studies involving humans were approved by Marondera University of Agricultural Sciences and Technology Research Board Ethics Committee. The studies were conducted in accordance with the local legislation and institutional requirements. Written informed consent for participation in this study was provided by the participants’ legal guardians/next of kin.

## Author contributions

KG: Conceptualization, Investigation, Writing – review & editing. MD: Conceptualization, Formal analysis, Investigation, Methodology, Writing – review & editing. ML: Conceptualization, Methodology, Writing – original draft, Writing – review & editing.
